# Early‐to‐Midlife Body Mass Index Trajectories and Obstructive Sleep Apnoea Risk 10 Years Later

**DOI:** 10.1111/resp.70002

**Published:** 2025-02-11

**Authors:** Yaoyao Qian, Jennifer L. Perret, Garun S. Hamilton, Michael J. Abramson, Caroline J. Lodge, Dinh S. Bui, Gulshan B. Ali, Anurika P. De Silva, Robert J. Adams, Bruce R. Thompson, Bircan Erbas, Eugene H. Walters, Chamara V. Senaratna, Shyamali C. Dharmage

**Affiliations:** ^1^ Allergy and Lung Health Unit, Melbourne School of Population and Global Health The University of Melbourne Melbourne Victoria Australia; ^2^ The Institute for Breathing and Sleep (IBAS) Melbourne Victoria Australia; ^3^ Department of Respiratory and Sleep Medicine Austin Hospital Melbourne Victoria Australia; ^4^ Monash Lung, Sleep, Allergy & Immunology Monash Health Melbourne Victoria Australia; ^5^ School of Clinical Sciences Monash University Melbourne Victoria Australia; ^6^ School of Public Health & Preventive Medicine Monash University Melbourne Victoria Australia; ^7^ Centre for Epidemiology and Biostatistics, Melbourne School of Population and Global Health The University of Melbourne Melbourne Victoria Australia; ^8^ Methods and Implementation Support for Clinical and Health Research Hub, Faculty of Medicine, Dentistry and Health Sciences The University of Melbourne Melbourne Victoria Australia; ^9^ Adelaide Institute for Sleep Health (AISH) Flinders University Adelaide South Australia Australia; ^10^ Melbourne School of Health Science The University of Melbourne Melbourne Victoria Australia; ^11^ School of Psychology & Public Health La Trobe University Melbourne Victoria Australia; ^12^ School of Medicine University of Tasmania Hobart Tasmania Australia

**Keywords:** BMI, body mass index trajectories, home sleep apnoea testing, longitudinal risk factors, obstructive sleep apnoea, screening questionnaires, sleep‐disordered breathing

## Abstract

**Background and Objective:**

While short‐term weight changes are known to influence obstructive sleep apnoea (OSA), the impact of body mass index (BMI) changes over the life course has been poorly documented. We examined the association between BMI trajectories from childhood to middle age and adult OSA, 10 years later.

**Methods:**

Five BMI trajectories were previously identified in the population‐based cohort Tasmanian Longitudinal Health Study (TAHS), using eight time‐point BMI from age 5 to 43 years. The primary outcome was probable OSA at 53 years, defined using STOP‐Bang questionnaire, with Berlin and OSA‐50 questionnaires used to ensure consistency of findings. Clinically significant diagnosed OSA was defined as self‐reported medical diagnosis or mild OSA with symptoms or moderate‐to‐severe OSA, using type‐4 sleep studies. Associations were examined using multivariable logistic regression.

**Results:**

Compared with the *average* BMI trajectory, the *child average‐increasing* (aOR = 5.28, 95% CI 3.38–8.27) and persistently *high* trajectories (aOR = 3.73, 2.06–6.74) were associated with increased risk of probable OSA. These associations were consistent when using clinically significant diagnosed OSA (*child average‐increasing* trajectory: aOR = 2.95, 1.30–6.72; *high* trajectory: aOR = 2.23, 0.82–6.09). Individuals belonging to the *low* trajectory were less likely than the *average* trajectory to have OSA. Notably, the *child high‐decreasing* trajectory was not associated with OSA.

**Conclusion:**

Physicians and the public should be aware of the potential risk of OSA in middle‐aged adults when BMI is high or continuously increasing from childhood to mid‐40s. Obese children who subsequently lose weight were not at higher risk of OSA in middle age—a novel and key finding.

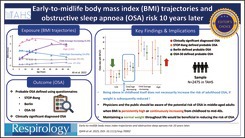

## Graphical abstract

**See related** editorial

**Summary**

- This is the first study to find that individuals with persistently “high” and “child average‐increasing” BMI trajectories from childhood to middle age were at increased risk of OSA, 10 years later.
- A novel finding is that obese children who subsequently lost weight were not associated with OSA in middle age.

## 1 Introduction

Obstructive sleep apnoea (OSA) causes repetitive upper airway obstruction during sleep and is estimated to affect one billion people worldwide [1, 2]. This high prevalence, together with adverse consequences of OSA including hypertension, stroke, diabetes, increased motor vehicle accidents, depression, and mortality [3, 4] represents a substantial public health burden [5].

While male sex, older age, craniofacial and upper airway abnormalities, menopause, and enlarged tonsils and adenoids are recognised risk factors for OSA [6, 7, 8], the epidemic of obesity over the past decades is likely to largely explain the increasing prevalence of OSA [9]. A cross‐sectional association between obesity and OSA in adults is well established [10, 11]. However, single‐time point measurements of body mass index (BMI) ignore obesity duration and the dynamic nature of weight changes [12, 13], so some studies have investigated weight changes between two‐time points [14]. For example, the Wisconsin Sleep Cohort Study found that weight gain from 46 to 50 years was associated with both the development and increasing severity of OSA in middle‐aged adults [14]. Yet, weight changes measured using two time points are insufficient to capture weight fluctuations over the life course, which can only be examined using longitudinal trajectories of BMI with data from multiple time points in life. The Bogalusa Heart Study used repeated BMI measurements to categorise childhood overweight patterns and investigated their association with OSA after 35 years [15], but the weight assessment was limited to childhood (4–18 years). Overall, no study to date has accounted for the impact of BMI changes over the life course on OSA [16]. Investigating the association of BMI trajectories from childhood to middle age with OSA later in life may identify the key weight patterns contributing to OSA and inform preventive strategies, as well as provide evidence for understanding the pathophysiological pathway of OSA development.

Using Tasmanian Longitudinal Health Study (TAHS) data collected across five decades of life, we previously characterised BMI trajectories from 5 to 43 years [13]. These data now provide a unique opportunity to investigate BMI trajectories over the life course and their association with OSA. This paper investigated the longitudinal associations of BMI trajectories from childhood to middle age (43 years) with obstructive sleep apnoea at age 53 years, 10 years later.

## 2 Methods

### 2.1 Study Design and Study Population

A detailed description of the methodology of TAHS has been previously published [17]. In brief, TAHS recruited 8583 Tasmanian schoolchildren born in 1961 who were, on average, 7 years old at baseline. More than 70% participants remained in Tasmania in the 2012 follow‐up. Subsequent follow‐ups were conducted at 13, 20, 31, 43, and 53 years of age. At the mean age of 53 years, all contactable participants from the original cohort were invited to participate in a survey that included OSA screening questionnaires and questions about previous OSA diagnoses. A random subgroup of participants attended home‐based type 4 sleep studies.

### 2.2 Exposure—Body Mass Index Trajectories

**FIGURE 1:**
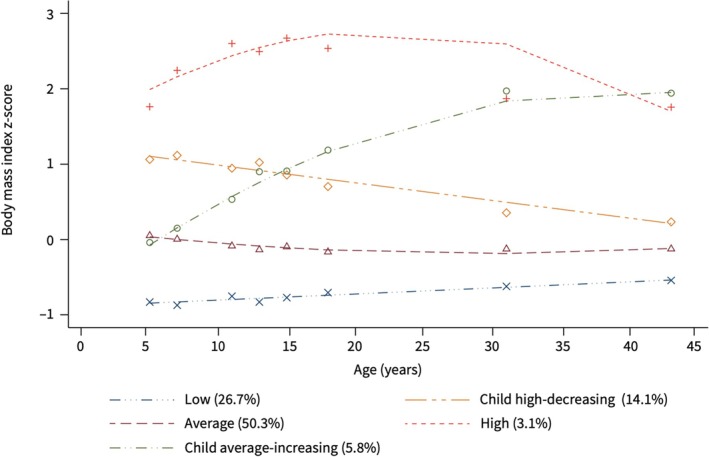
Body mass index (z‐score) trajectories classes across ages from 5 to 43 years. Five BMI trajectories were identified among a total of 4194 participants with 8 repeated measurements at 5–6, 7, 10–11, 13, 14–15, 20, 30, and 43 years using Tasmanian Longitudinal Health Study and school medical records. Reproduced with permission of the ERS 2024: *European Respiratory Journal* 60, (2022): 2102286; DOI: 10.1183/13993003.02286‐2021.

Information on BMI trajectories within TAHS has been published [13]. Weight/height information was available for a total of 8‐time points: three from school medical records (at ages 5–6, 10–11, and 14–15 years) and five from TAHS follow‐ups (at ages 7, 13, 20, 31, and 43 years), and BMI was calculated and converted to age‐ and sex‐specific z‐scores. Group‐based trajectory modelling (GBTM), based on maximum likelihood estimation to determine the best‐fitting model was used to group individuals whose BMI z‐score changes (5–43 years) showed similar patterns over time [18]. Five distinct BMI trajectories were identified and named based on their shapes: *average, low, child high‐decreasing, child average‐increasing*, and *high* trajectories (Figure 1) [13]. The *average* trajectory had a BMI z‐score of around 0 persistently. The *child average‐increasing* trajectory had a continuous increase in BMI z‐score from childhood to middle age and its z‐score exceeded that of the *high* trajectory at around 40 years. Despite a high weight in childhood, the BMI z‐score of the *child‐high decreasing* trajectory decreased and approached the *average* trajectory gradually (from 5 to 43 years; Figure 1).

### 2.3 Primary Outcome—Probable OSA

In large population‐based studies, validated screening questionnaires that detect probable OSA are commonly used due to the logistical difficulties and heavy financial burden of sleep studies [19, 20]. Our primary outcome is probable OSA defined using the STOP‐Bang questionnaire, which is a self‐report screening tool comprising 8 items evaluating ***S*** noring, ***T*** iredness during the daytime, ***O*** bserved stopped breathing during sleep, high blood ***P*** ressure, ***B*** MI, ***A*** ge, ***N*** eck circumferences, and ***G*** ender [21] and has been found to perform well in detecting OSA in both clinical and population‐based settings [22]. The cut‐off level of STOP‐Bang can be increased to achieve higher specificity but with a lower sensitivity as a trade‐off [23]. The Berlin (BQ) and OSA‐50 questionnaires are other screening tools used to detect probable OSA at 53 years [24, 25], and were applied to assess the consistency of the findings. All three questionnaires have standard cut‐off scores (≥ 3 for STOP‐Bang [21], ≥ 2 for BQ [24] and ≥ 5 for OSA‐50 [25]). However, when revalidated within TAHS, the optimal cut‐off score was ≥ 5 for STOP‐Bang, ≥ 6 for OSA‐50 questionnaire, and ≥ 2 for BQ, which was used in this analysis [23].

### 2.4 Secondary Outcome—Clinically Significant Diagnosed OSA

Information on medically diagnosed OSA was collected using a self‐reported survey question at age 53 years, “Has your doctor ever told you that you have or had obstructive sleep apnoea?”. In the 53‐year follow‐up in TAHS, a random sample of 772 subjects from respiratory laboratory attendees were invited for home sleep studies, and in total 10% (371/3609) of participants in this follow‐up completed an objective sleep test using limited channel home sleep apnoea testing (HSAT). Portable ApneaLink (ResMed, Bella Vista, Australia) devices, which recorded information on pulse rate, oxygen desaturation, nasal airflow and snoring during sleep, were used to perform the HSAT [26]. Oxygen desaturation index (ODI_3%_), defined as average numbers of desaturation events (decrease in the mean oxygen saturation ≥ 3%) per hour, from ApneaLink has been validated against laboratory polysomnography (PSG) and showed excellent utility for detecting moderate‐to‐severe OSA with high sensitivity (96.4%) and specificity (88.2%) [27]. Also, in type 4 sleep studies ODI is a more robust signal and more sensitive than the flow‐based apnoea hypopnoea index (AHI) in capturing OSA cases [26]. Therefore, ODI was used to define objectively determined OSA in this study [26, 27].

Mild OSA is common but not treated unless symptomatic (e.g., excessive daytime sleepiness), while moderate‐to‐severe OSA is an indication for treatment regardless of symptoms [28]. Therefore, we defined clinically significant diagnosed OSA as either (1) self‐reported OSA diagnosis or (2) mild OSA (5 ≥ ODI > 15) with excessive daytime sleepiness (Epworth sleepiness scale ≥ 8), or (3) moderate‐to‐severe OSA (ODI ≥ 15), the latter two based on type‐4 ApneaLink sleep studies [23, 29].

### 2.5 Statistical Analysis

All statistical analyses were conducted using STATA version 16.0 (Stata Corporation, College Station, Texas, USA). The descriptive data were reported as numbers and percentages or means and standard deviations (SDs). The missing values for medically diagnosed OSA and sleep studies were handled using multiple imputations [30] (MI; see Appendix S1 for details). Multivariable logistic regression models were fitted to examine the associations between BMI trajectories from 5 to 43 years and probable OSA, defined mainly using STOP‐Bang, but also using Berlin, and OSA‐50 for consistency. Models were repeated using clinically significant diagnosed OSA in place of probable OSA. As STOP‐Bang contains obesity assessment, we conducted a sensitivity analysis by restricting the analysis to those who were not assessed as obese (BMI ≤ 35 kg/m^2^). All associations were adjusted for a minimally sufficient set of confounders (adult physical activity, adult smoking, childhood second‐hand smoking status, and adult alcohol consumption; see Appendix S2 for details), identified using a directed acyclic graph (DAG), which was developed based on the known literature and content expertise (Figure S1). To test the temporal association of single‐time BMI with OSA, regression models were used to examine the association of BMI at ages 7 and 43 years (see Appendix S3 for details) with probable OSA at 53 years, separately. The results were reported as odds ratios (ORs) with 95% confidence intervals (CI). Interactions between BMI trajectories and potential effect modifiers, that is, biological sex, current asthma status, current smoking status, current diet, and current physical activity at 53 years (see Appendix S2 for details), were tested using the likelihood ratio tests (LRTs) and strata‐specific estimates were only reported if the *p* value was consistently < 0.1 when using three different questionnaires.

## 3 Results

**FIGURE 2:**
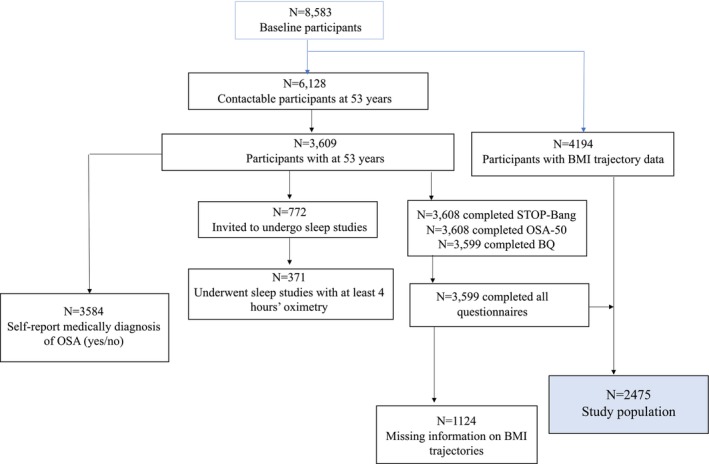
Flow diagram of study population selection in the Tasmanian Longitudinal Health Study (TAHS). BMI, body mass index; BQ, Berlin questionnaire; ESS, Epworth sleepiness scale; OSA, obstructive sleep apnoea.

Of 3609 participants at mean age 53 years, 2475 had data on BMI trajectory and all three OSA screening questionnaires, and therefore comprised the study population (Figure 2). This group had a slighter lower prevalence of current smokers and had a higher socioeconomic status compared with those excluded (*n* = 1134) from the analysis (Table S1).

Half of the study population (50.3%) belonged to the *average* BMI trajectory, which was treated as the reference group, followed by the *low* (27.4%), *child high‐decreasing* (14.3%), *child average‐increasing* (5.2%) and *high* (2.8%) trajectories (Table 1). The highest mean BMI at 53 years was in the *child average‐increasing* trajectory (mean BMI ± SD: 37.3 ± 6.2 kg/m^2^) and persistently *high* trajectory (mean BMI ± SD: 37.2 ± 8.1 kg/m^2^; Table 1). At 43 years, despite a continuous decrease in BMI z‐scores, individuals belonging to the *child high‐decreasing* trajectory were still overweight (mean BMI ± SD: 27.4 ± 3.3 kg/m^2^). Our group has demonstrated that the prevalence of maternal smoking and maternal asthma was higher in the *child average‐increasing* and *high* trajectories, and the prevalence of small for gestation age was higher in the *low* trajectory, compared to the *average* trajectory [13].

**TABLE 1:** Characteristics of the study population in each BMI trajectory group (*N* = 2475).

Characteristics *N* (%)	Average (*n* = 1245, 50.3%)	Low (*n* = 679, 27.4%)	Child high‐decreasing (*n* = 353, 14.3%)	Child average‐increasing (*n* = 129, 5.2%)	High (*n* = 69, 2.8%)	Total (*n* = 2475)
Country of birth						
Australia	1212 (97.4%)	655 (96.5%)	342 (96.9%)	128 (99.2%)	67 (97.1%)	2404 (97.1%)
Sex						
Female	613 (49.2%)	342 (50.4%)	188 (53.3%)	78 (60.5%)	42 (60.9%)	1263 (51.0%)
Characteristics at 53 years						
BMI, kg/m^2^‐mean (± SD)						
	28.0 (4.5)	25.4 (3.8)	29.8 (4.7)	37.3 (6.2)	37.2 (8.1)	28.3 (5.5)
Current residence						
Tasmania	957/1234 (77.6%)	527/674 (78.2%)	261/347 (75.2%)	107/127 (84.3%)	54 (78.3%)	1906/2451 (77.8%)
Victoria	77/1234 (6.2%)	52/674 (7.7%)	26/347 (7.5%)	7/127 (5.5%)	7 (10.1%)	169/2451 (6.9%)
New South Wales	47/1234 (3.8%)	22/674 (3.3%)	13/347 (3.8%)	1/127 (0.8%)	1 (1.5%)	84/2451 (3.4%)
Others	153/1234 (12.4%)	73/674 (10.8%)	47/347 (13.5%)	12/127 (9.5%)	7 (10.1%)	292/2451 (11.9%)
Asthma status						
Current asthma	188/1243 (15.1%)	100 (14.7%)	50/352 (14.2%)	30 (23.3%)	16/68 (23.5%)	384/2471 (15.5%)
Shortness of breath at rest in the past year						
Yes	98/1234 (7.9%)	45/670 (6.7%)	25/350 (7.1%)	7 (5.4%)	9 (13.0%)	184/2452 (7.5%)
Shortness of breath after exercise in the past year						
Yes	224/1234 (18.2%)	114/673 (16.9%)	57/350 (16.3%)	39 (30.2%)	15 (21.7%)	449/2455 (18.3%)
Wheezing and whistling in the chest in the past year						
Yes	248/1240 (20.0%)	114/673 (16.9%)	82/351 (23.4%)	37/128 (28.9%)	19 (27.5%)	500/2461 (20.3%)
Smoking status						
Never	542/1245 (43.5%)	310/678 (45.7%)	152 (43.1%)	54 (41.9%)	35 (50.7%)	1093/2474 (44.2%)
Past smoker	488/1245 (39.2%)	264/678 (38.9%)	131 (37.1%)	58 (45.0%)	23 (33.3%)	964/2474 (39.0%)
Current smoker	215/1245 (17.3%)	104/678 (15.3%)	70 (19.8%)	17 (13.2%)	11 (15.9%)	417/2474 (16.9%)
Current physical activities						
Low	224/1069 (21.0%)	127/589 (21.6%)	60/311 (19.3%)	36/107 (33.6%)	20/59 (33.9%)	467/2135 (21.9%)
Moderate	364/1069 (34.1%)	216/589 (36.7%)	95/311 (30.6%)	32/107 (29.9%)	22/59 (37.3%)	729/2125 (34.2%)
High	481/1069 (45.0%)	246/589 (41.8%)	156/311 (50.2%)	39/107 (36.5%)	17/59 (28.8%)	939/2125 (44.0%)
Amount of pure alcohol per week, grams—mean (± SD)						
	89.0 (109.7)	88.9 (113.1)	84.1 (101.9)	52.9 (149.6)	42.3 (53.8)	85.1 (111.3)
Sleep studies (*n* = 254)						
Apnoea‐hypopnoea index (events/h)						
AHI < 5	5/127 (44.1%)	35/66 (53.0%)	17/33 (51.5%)	6/16 (37.5%)	3/12 (25.0%)	117/254 (46.1%)
15 > AHI ≥ 5	53/127 (41.7%)	22/66 (33.3%)	15/33 (45.5%)	4/16 (25.0%)	7/12 (58.3%)	101/254 (39.8%)
AHI ≥ 15	18/127 (14.2%)	9/66 (13.6%)	1/33 (3.0%)	6/16 (37.5%)	2/12 (16.7%)	36/254 (14.2%)
Oxygen desaturation index (events/h)						
ODI < 5	40/127 (31.5%)	17/66 (25.8%)	11/33 (33.3%)	2/16 (12.5%)	2/12 (16.7%)	72/254 (28.4%)
15 > ODI ≥ 5	56/127 (44.1%)	36/66 (54.6%)	14/33 (42.4%)	5/16 (31.3%)	4/12 (33.3%)	115/254 (45.3%)
ODI ≥ 15	31/127 (24.4%)	13/66 (19.7%)	8/33 (24.2%)	9/16 (56.3%)	6/12 (50.0%)	67/254 (26.4%)
Probable OSA						
STOP‐Bang (≥ 5)	168 (13.5%)	66 (9.7%)	48 (13.6%)	48 (37.2%)	21 (30.4%)	351 (14.2%)
Berlin (≥ 2)	438 (35.2%)	167 (24.6%)	144 (40.8%)	86 (66.7%)	36 (52.2%)	871 (35.2%)
OSA‐50 (≥ 6)	448 (36.0%)	190 (28.0%)	136 (38.5%)	75 (58.1%)	26 (37.7%)	875 (35.4%)

Abbreviations: AHI, apnoea hypopnoea index; BMI, body mass index; ODI, oxygen desaturation index; OSA, obstructive sleep apnoea; SD, standard deviation.

The mean increase in BMI from 5 to 43 years varied between 9.4 and 21.3 kg/m^2^ across all trajectories. During the transition from early adulthood to middle age (20 to 43 years), the BMI increase was highest for the *child average‐increasing* trajectory (10.2 kg/m^2^, with an annual mean increase of 0.44 kg/m^2^), much higher than that for the other trajectories (BMI increases ranged from 1.8 to 4.3 kg/m^2^; Figure S2).

### 3.1 Associations of BMI Trajectories With Probable OSA

**FIGURE 3:**
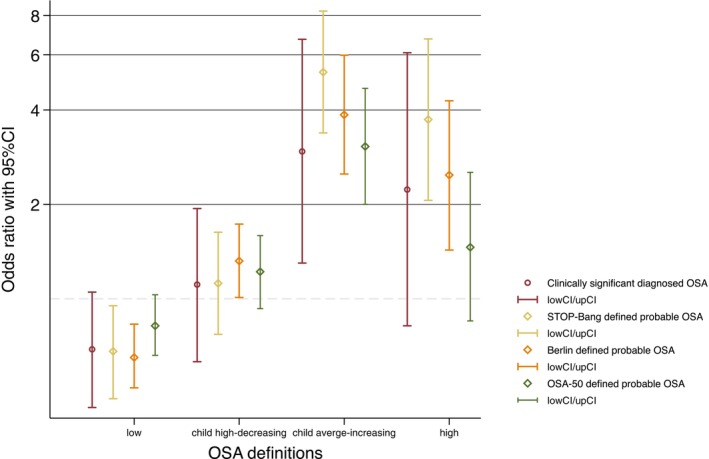
The adjusted# associations of each BMI trajectory with clinically significant diagnosed OSA and probable OSA with different definitions. The reference group is the average trajectory. # Odds ratios and 95% confidence intervals from logistic regression models adjusting for adult physical activity, childhood second‐hand smoking, adult alcohol consumption, and adult smoking. BMI, body mass index; CI, confidence interval; OSA, obstructive sleep apnoea.

When using the STOP‐Bang questionnaire to define probable OSA, compared with the *average* trajectory, the *child average‐increasing* trajectory was associated with the highest risk of probable OSA (aOR = 5.28, 95% CI 3.38–8.27). Individuals belonging to the *high* trajectory were more likely (aOR = 3.73, 95% CI 2.06–6.74) to have probable OSA, while those belonging to the *low* trajectory were less likely (aOR = 0.68, 95% CI 0.48–0.95) to have probable OSA (Table 2). Consistent findings were observed when Berlin and OSA‐50 questionnaires were used to define probable OSA in place of STOP‐Bang (Figure 3; Table S2).

**TABLE 2:** The associations of BMI trajectories with probable OSA defined using the STOP‐Bang questionnaire (*N* = 2475).

BMI‐trajectories	Prevalence (n/N)	Unadjusted, OR (95% CI), *p* value	Adjusted, aOR^a^ (95% CI), *p* value
Average	13.5% (168/1245)	—	—
Low	9.7% (66/679)	**0.69 (0.51, 0.93), *p* = 0.016**	**0.68 (0.48, 0.95), *p* = 0.024**
Child high‐decreasing	13.6% (48/353)	1.01 (0.71, 1.42), *p* = 0.960	1.12 (0.77, 1.63), *p* = 0.544
Child average‐increasing	37.2% (48/129)	**3.80 (2.57, 5.62), *p* < 0.001**	**5.28 (3.38, 8.27), *p* < 0.001**
High	30.4% (21/69)	**2.80 (1.64, 4.80), *p* < 0.001**	**3.73 (2.06, 6.74), *p* < 0.001**

*Note*: The estimates were in bold if *p* value < 0.05.

Abbreviations: BMI, body mass index; CI, confidence interval; OR, odds ratio; OSA, obstructive sleep apnoea.

^a^ Odds ratios and 95% confidence intervals from logistic regression models adjusting for adult physical activity, childhood second‐hand smoking, adult alcohol consumption, and adult smoking.

When the analysis was restricted to those with BMI ≤ 35 kg/m^2^, the *child average‐increasing* trajectory continued to be associated with higher risk of probable OSA defined using STOP‐Bang (aOR = 2.58, 95% CI 1.14–5.87; Table S3).

### 3.2 Associations of BMI Trajectories With Clinically Significant Diagnosed OSA

Compared with the *average* trajectory, the *child average‐increasing* trajectory was consistently associated with higher risk of clinically significant diagnosed OSA (aOR = 2.95, 95% CI 1.30–6.72). We also observed consistent modest evidence that the *high* trajectory was associated with clinically significant diagnosed OSA (aOR = 2.23, 95% CI 0.82–6.09; Table 3).

**TABLE 3:** The association of BMI trajectories with clinically significant diagnosed OSA (*N* = 2475).

	Clinically significant diagnosed OSA (15 > ODI ≥ 5 plus ESS ≥ 8 or ODI ≥ 15 or diagnosed OSA)	
BMI‐trajectories	Unadjusted, OR (95% CI), *p* value	Adjusted^a^, OR (95% CI), *p* value
Average	—	—
Low	0.70 (0.47, 1.03), *p* = 0.073	0.69 (0.45, 1.05), *p* = 0.080
Child high‐decreasing	1.07 (0.63, 1.81), *p* = 0.811	1.11 (0.63, 1.94), *p* = 0.722
Child average‐increasing	**2.98 (1.35, 6.51), *p* = 0.007**	**2.95 (1.30, 6.72), *p* = 0.010**
High	2.29 (0.89, 5.84), *p* = 0.084	2.23 (0.82, 6.09), *p* = 0.115

*Note*: This table is based on multiply imputed data. The estimates were in bold if *p* value < 0.05.

^a^ Odds ratios and 95% confidence intervals from logistic regression models adjusting for adult physical activity, childhood second‐hand smoking, adult alcohol consumption, and adult smoking.

Abbreviations: BMI, body mass index; CI, confidence interval; OR, odds ratio; OSA, obstructive sleep apnoea.

### 3.3 Effect Modifications

There was some evidence that current asthma modified the association between BMI trajectories and probable OSA defined using STOP‐Bang, and current smoking status modified the association between BMI trajectories and probable OSA defined using the OSA‐50 questionnaire (Table S4). However, no consistent effect modifier was observed across three screening questionnaires.

### 3.4 Associations of BMI at 7 and 43 Years With Probable at 53 Years

Overweight or obesity at 7 years were not consistently associated with probable OSA defined using STOP‐Bang, BQ or OSA‐50 (Table S5). However, both overweight (25 ≤ BMI < 30 kg/m^2^) and obesity (BMI ≥ 30 kg/m^2^) at age 43 years were consistently associated with probable OSA at 53 years (Table S6).

## 4 Discussion

This is the first population‐based study that investigated the temporal association between BMI trajectories from childhood to middle age and OSA, 10 years later. More importantly, our study is novel as it investigated two BMI trajectories that have not been previously examined in relation to OSA: persistently *low* and *child high‐decreasing* trajectories. The *low* trajectory was associated with decreased risk of probable OSA. Notably, the null association between the *child high‐decreasing* trajectory and OSA suggests that childhood obesity did not increase the risk of OSA in middle age, if weight was lost over time. Additionally, similar to the findings from previous short‐term studies, we found that the *child average‐increasing* trajectory, a weight pattern with a relatively healthy BMI in childhood but increasing rapidly thereafter, was consistently associated with the highest risk of both probable OSA and clinically significant diagnosed OSA in middle age. The persistently *high* trajectory group was also associated with probable OSA and clinically significant diagnosed OSA. Together, our findings provide new insights into the associations between weight changes over 4 decades and adult OSA.

Some potential mechanisms have been proposed for the association between obesity and OSA. Fat deposition in the tongue and/or around the pharynx can narrow and alter the anatomy of the upper airway, leading to the reduction or cessation of airflow [31]. Furthermore, OSA has been independently associated with abdominal visceral adipose tissue deposition, which may reduce lung volumes [32, 33]. Leptin resistance, which commonly occurs in obesity, may destabilise respiratory control during sleep, contributing to OSA in some people [34, 35]. Other factors, such as hormonal imbalances and metabolic dysfunction, may interact with increased fat deposition and inflammation, potentially predisposing individuals to OSA [36]. On the other hand, weight loss may improve OSA by reducing the abdominal fat and then improving airway traction or by reducing several upper airway soft tissues [37, 38], both of which could prevent upper airway collapse during sleep.

To minimise the influence of obesity assessment contained within the STOP‐Bang, we performed a sensitivity analysis, restricted to individuals whose BMI ≤ 35 kg/m^2^ at 53 years. The association between the *child average‐increasing* trajectory and OSA was consistently observed in this setting. Furthermore, the robustness of our findings was supported by the consistent relationship between *child average‐increasing* and persistently *high* trajectories and clinically significant diagnosed OSA, our secondary outcome. The results remained unchanged using different OSA definitions.

Individuals who belonged to the *low* trajectory with persistently low weight throughout life were less likely to have probable OSA in middle age compared with the average trajectory. Notably, despite being classified as the *low* trajectory, they were still within the normal weight range with the mean BMI z‐score ranging from −1 to −0.5 at all time points [13]. Hence, along with a healthy lifestyle, maintaining a relatively low but healthy weight throughout life should be encouraged to reduce the risk of OSA.

No previous study has investigated the impact of life course BMI trajectories on OSA. However, there are studies that have investigated the association between shorter‐term weight gain and OSA, such as weight gain in children/adolescents or middle‐aged adults only. The Bogalusa Heart Study identified four overweight patterns in childhood and adolescence (4–18 years) with 10 repeated measurements [15]. They found that both incident and persistent overweight in this period were linked to BQ‐defined probable OSA at age 43 years [15]. Another study with 10‐year follow‐up in Hongkong also found an increase in BMI z‐score from childhood (9.8 ± 1.8 years) to young adulthood was associated with increased risk of new‐onset OSA determined by polysomnography by mean age 20.2 years [39]. The Wisconsin Sleep Cohort Study found that gaining weight from 46 to 50 years was associated with the development of moderate‐to‐severe OSA at 50 years defined by PSG [14]. Overall, despite different age groups, these results of short‐term studies were consistent with ours and confirmed the association between weight gain and higher risk of OSA. The novelty of our study compared to these short‐term studies is that our *child average‐increasing* and *high* (BMI) trajectories were derived from childhood to middle age between the first and fifth decade of life, which provided more robust evidence to show that life course weight gain and persistent overweight increased the risk of OSA. The *child average‐increasing* trajectory showed the highest BMI increase (mean 10.2 kg/m^2^) from early adulthood to middle age (20 to 43 years), which suggested that OSA risk increased particularly with more rapid weight gain.

Most children (90.1%) in our study were within the normal weight at age 7 years, and BMI at this age per se was not associated with OSA at age 53 years. Some studies have found that childhood obesity was associated with adult OSA [39]. This disparity emphasises the necessity to examine whether the impact of BMI on OSA is immediate or enduring [40]. Our BMI trajectories from childhood to middle age, robustly developed using eight measurements, provided the opportunity to examine the impact of dynamic changes in weight spanning over 40 years on OSA 10 years later. In addition, we found temporal associations of overweight and obesity at 43 years with OSA at 53 years. But, interestingly, despite being averagely overweight (mean BMI 27.4 kg/m^2^) at 43 years, individuals belonging to the *child high‐decreasing* trajectory were not at higher risk of OSA compared with the *average* group, highlighting the importance and benefit of weight loss across the life course for obese individuals.

Our study has several strengths: TAHS is a whole population cohort with a long follow‐up time over five decades, which, together with the advanced statistical methods (i.e., GBTM), has enabled the establishment of distinct life‐course BMI trajectories. This was not possible in previous studies that investigated the BMI‐OSA association. By assessing OSA 10 years after establishing BMI trajectories, we were able to examine the temporal association and avoid reverse causation. Also, the use of multiple OSA definitions showed consistent results, further supporting the robustness of the findings.

However, there were some limitations in our study: A limitation of our study was the reliance on the STOP‐Bang questionnaire to define probable OSA as the primary outcome. The high specificity but poor sensitivity of this definition may have led to the underestimation of OSA case [23]. However, any misclassification was likely to be non‐differential, and our consistent findings using multiple OSA definitions support the robustness of the observed associations. Although PSGs, the gold standard for OSA diagnosis, were not performed, we used ODI_3%_ to indicate objectively determined OSA, which has been validated as a robust signal for type‐4 sleep studies. While we used either sleep study results or self‐reported diagnosis to define clinically significant diagnosed OSA, only a randomly selected subgroup of the cohort underwent sleep studies. This might have led to non‐differential misclassification and underestimation of the association. However, we have used multiple imputation to handle the missing values. MI may introduce some bias to the results, but the association between *child average‐increasing* trajectory and OSA were consistently observed using different OSA definitions. OSA and its treatment were only assessed at age 53 years. Therefore, the effect of BMI trajectories on the incidence and progression of OSA could not be inferred. The potential confounding by OSA treatment, which may have a beneficial effect on BMI trajectory, could not be excluded but would likely move the results toward the null without affecting the overall strength. Additionally, the lack of data on childhood physical activity may have resulted in residual confounding. As TAHS participants were almost exclusively Caucasian [17], the finding should be generalised to other ethic populations with caution.

Our findings have multiple implications for clinical practice and future research. We have provided novel insights into the effect of life‐course weight patterns on OSA in middle age. Healthcare providers, including general practitioners (GPs), can use this information when evaluating whether a patient has susceptibility to OSA on these grounds. Individuals with an average weight in childhood, but experiencing continuous and substantial increases into and during adulthood should be aware of the increased risk of OSA. An at least 0.44 kg/m^2^ annual mean increase in adulthood BMI might flag a greater susceptibility to OSA. Given the impact of the *high* BMI trajectory on OSA, GPs and the public should also be aware of the possibility of OSA when there is a persistently high BMI throughout life. However, the highest risk for OSA may be conferred by a more rapid proportional weight gain. Moreover, our findings provide an important public health message that being obese in childhood may not increase the risk of OSA in middle age, if weight is subsequently lost toward a healthier BMI. As modest weight loss has been shown to reduce the severity of OSA, future studies are recommended to quantify the minimum weight loss needed to reduce the risk of OSA for obese children [41]. Developing a novel prediction model for OSA, which takes into account weight changes over the life course rather than single‐point or short‐term weight changes, is encouraged in the future.

In conclusion, our large, prospective population‐based study was the first to find that individuals with continuously increasing and persistently high BMI from childhood to middle age are at higher risk of having OSA later in life. Being obese in childhood itself does not necessarily increase the risk of adulthood OSA, if weight is subsequently reduced. Maintaining a normal weight throughout life would be beneficial in reducing the risk of OSA.

## Author Contributions

**Yaoyao Qian:** formal analysis (lead), investigation (equal), methodology (lead), visualization (lead), writing – original draft (lead), writing – review and editing (lead). **Jennifer L. Perret:** conceptualization (equal), funding acquisition (supporting), investigation (supporting), project administration (supporting), supervision (equal), writing – review and editing (equal). **Garun S. Hamilton:** conceptualization (equal), funding acquisition (supporting), investigation (supporting), project administration (supporting), supervision (equal), writing – review and editing (equal). **Michael J. Abramson:** funding acquisition (supporting), investigation (supporting), writing – review and editing (supporting). **Caroline J. Lodge:** funding acquisition (supporting), investigation (supporting), writing – review and editing (supporting). **Dinh S. Bui:** investigation (supporting), writing – review and editing (supporting). **Gulshan B. Ali:** formal analysis (supporting), writing – review and editing (supporting). **Anurika P. De Silva:** formal analysis (supporting), methodology (supporting), writing – review and editing (supporting). **Robert J. Adams:** investigation (supporting), writing – review and editing (supporting). **Bruce R. Thompson:** investigation (supporting), writing – review and editing (supporting). **Bircan Erbas:** investigation (supporting), writing – review and editing (supporting). **Eugene H. Walters:** funding acquisition (supporting), investigation (supporting), writing – review and editing (supporting). **Chamara V. Senaratna:** conceptualization (equal), investigation (equal), project administration (equal), supervision (lead), writing – review and editing (equal). **Shyamali C. Dharmage:** conceptualization (equal), funding acquisition (lead), investigation (equal), project administration (lead), supervision (lead), writing – review and editing (supporting).

## Ethics Statement

TAHS was approved by the Human Research Ethics Committee of the University of Melbourne (ethics ID number: RES‐22‐0000‐046A). All participants provided written informed consent.

## Conflicts of Interest

Bircan Erbas is a Statistical Review Board member of Respirology and co‐author of this article. She was excluded from all editorial decision‐making related to the acceptance of this article for publication.

M.J.A. holds investigator‐initiated grants for unrelated research from Boehringer‐Ingelheim, Pfizer, GlaxoSmithKline and Sanofi. He has undertaken an unrelated consultancy for Sanofi and received a speaker's fee from G.S.K. E.H.W., C.J.L., S.C.D., J.L.P. and D.S.B hold an investigator‐initiated grant from GlaxoSmithKline for unrelated research. S.C.D., J.L.P., C.J.L. are supported by the National Health and Medical Research Council of Australia (NHMRC) of Australia. S.C.D. has investigator‐initiated grant from Sanofi. J.L.P. is financially supported by the Australian Asthma Foundation, Craig Clifford Medical Trust, Helen McPherson Trust. G.S.H. receives equipment to support research from ResMed and Air Liquide Healthcare. The other co‐authors declare that they have no competing interests.

## Supporting information


**Data S1.** Supporting Information.

**Visual Abstract** Early‐to‐midlife body mass index (BMI) trajectories and obstructive sleep apnoea (OSA) risk 10 years later

## Data Availability

Any additional information regarding data in this manuscript are available from the corresponding author.
