# Supplementary material for: Early‐to‐Midlife Body Mass Index Trajectories and Obstructive Sleep Apnoea Risk 10 Years Later
Source: Respirology. 2025 Feb 11;30(5):435–45. doi: 10.1111/resp.70002 (PMC12060751; doi:10.1111/resp.70002)
Supplement: Supplementary file 1 — Data S1. Supporting Information. [file RESP-30-435-s001.docx]

**Supporting information**

**Appendix S1**

**Multiple imputation (MI) for missing data**

At age 53 years, participants were randomly selected to undergo type-4 sleep studies using ApneaLink^TM^, and 371 completed the test, leading to 90% missing data in objectively measured OSA. There was also 5% missing data in medically diagnosed OSA. We observed some differences, albeit minimal in characteristics between those with and without missing data. Missing data in medically diagnosed OSA and objectively measured OSA from sleep studies were multiply imputed, using chained equations. Missing data in the outcome- clinically significant diagnosed OSA were imputed passively within the imputation algorithm, using the imputed medically diagnosed OSA and sleep studies (1). Imputation models included all variables in the analysis model, BMI trajectories, physical activities, childhood second-hand smoking, adult smoking and alcohol drinking, and additional auxiliary variables, gender and BMI at 53 years (2). 90 imputations were used as there was approximately 90% missing data (3). Studies have shown to produce valid inferences even with a large proportion of missing data (up to 90%), given that the imputation model is correctly specified, and data are missing at random (4). Further analysis to assess sensitivity to missingness not at random was beyond the scope of our study.

**Appendix S2**

**Covariates at definitions**

**The information about these variables were collected by questionnaires in the Tasmanian Longitudinal Health Study (TAHS)**

- Adult physical activity at 53 years was defined using the International Physical Activity Questionnaire-Short Form (IPAQ-short).
- Current diet at 53 years was assessment using a Dietary Fat Questionnaire.
- Adult ever smoking was defined by asking the participants at 53 years “In your lifetime, have you smoked at least 100 cigarettes or equal amounts of cigars, pipes or any tobacco product?”.
- Adult current smoking was defined as affirmative responses to “In your lifetime, have you smoked at least 100 cigarettes or equal amounts of cigars, pipes or any tobacco product?” and “Do you currently smoke (within the last 4 weeks)?” or answering “Yes” to the first question and having valid responses to “On average how much (per day/week/month) do you currently smoke?”
- Current asthma status at 53 years was defined as using questions about 1) Asthma-related symptoms within the last 12 months, or 2) The use of asthma medicines within the last 12 month, or 3) The use of asthma-related healthcare services within the last 12 months.
- Childhood second-hand smoking was if the participants answered “Yes” to at least one of the questions “Did your father/mother smoke during the first year of your life?” and “Did your father/mother smoke when you were aged 1 to 4 years?” and “Did your father/mother smoke when you were aged 5 to 15 years?”.
- Adult alcohol consumption at 53 years was defined by asking the participants “Over the last 12 months, how often did you drink beer, wine and/or spirits?” and “Over the last 12 months, on days when you were drinking, how many glasses of beer, wine and/or spirits did you usually drink?”. The total amount of alcohol per week was calculated for participants.

**Appendix S3**

**Childhood weight at 7 years**

Weight in adulthood was categorised into normal weight, overweight and obesity using zbmicat() function in STATA, which used BMI cut-offs recommended by the Childhood Obesity Working Group of the International Obesity Taskforce (5).

**Adulthood weight at 43 years**

Weight in adulthood was categorised into underweight (BMI>18.5kg/m^2^), normal weight (18.5$\leq$BMI<25 kg/m^2^), overweight (25$\leq$BMI<30 kg/m^2^) and obesity (25 kg/m^2^$\leq$BMI) based on their body mass index.

**Table S1. Characteristics of study population and those who participated in the 6^th^ decade follow-up but were excluded from the analysis**

| **Characteristics**  **N (%)** | **Study population**  **(n=2475)** | **Excluded study population**  **(n=1134)** | **All participants in the 6^th^ decade follow-up**  **(n=3609)** |
| --- | --- | --- | --- |
| **BMI, kg/m^2^-mean (SD)*** | 28.3 (5.5) | 28.9 (6.0) | 28.5 (5.6) |
| **Sex** | | |  |
| Male | 1212 (49.0%) | 555 (48.9%) | 1767 (49.0%) |
| **Smoking status** | | |  |
| Never | 1093/2474 (44.2%) | 462/1132 (40.8%) | 1555 (43.1%) |
| Past smoker | 964/2474 (39.0%) | 457/1132 (40.4%) | 1421 (39.4%) |
| Current smoker | 417/2474 (16.9%) | 213/1132 (18.8%) | 630 (17.5%) |
| **Physical activity levels*** | | |  |
| Low | 467/2135 (21.9%) | 249/941 (26.5%) | 716/3076 (23.3%) |
| Moderate | 729/2135 (34.2%) | 292/941 (31.0%) | 1021/3076 (33.2%) |
| High | 939/2135 (44.0%) | 400/941 (42.5%) | 1339/3076 (43.5%) |
| **Adult SES levels** *, ϕ | | | |
| First class (highest) | 1662/2441 (68.1%) | 688/1117 (61.6%) | 2350/3558 (66.1%) |
| Second class | 447/2441 (18.3%) | 222/1117 (19.9%) | 669/3558 (18.8%) |
| Third class | 332/2441 (13.6%) | 207/1117 (18.5%) | 539/3558 (15.2%) |
| **STOP-Bang** | | | |
| High risk  (STOP-Bang$\geq$5) | 351 (14.2%) | 180/1133 (15.9%) | 531/3608 (14.7%) |
| **OSA-50** | | | |
| High risk  (OSA-50$\geq$6) | 875 (35.4%) | 431/1133 (38.0%) | 1306/3608 (36.2%) |
| **Berlin questionnaire*** | | | |
| High risk  ($\geq$2 domains positive) | 871 (35.2%) | 438/1124 (39.0%) | 1309/3599 (36.4%) |

ϕ Adult SES levels were assessed using adult occupation according to the International Standard Classification of Occupations (ISCO-88) four-digit classification. The first class includes Manager or administrator, Professional, Associate professional, Tradesperson or related worker, Advanced clerical or service worker. The second class includes Intermediate clerical, sales, service worker and Intermediate production or transport worker. The third class includes Elementary clerical, sales or service worker, Labourer or related worker and House person

* The characteristics were different between the study population and those who were excluded from the analysis using t-test or chi-square test with a p-value <0.05.

Abbreviations: SD=Standard deviation; SES=Socioeconomic status

**Table S2. The association of BMI trajectories with probable OSA defined by Berlin and OSA-50 questionnaires using optimal cut-offs (n=2475).**

|  | **Berlin questionnaires** | | **OSA-50 questionnaire** | |
| --- | --- | --- | --- | --- |
| **BMI-trajectories** | **Prevalence (n/N)** | **aOR# (95% CI), p-value** | **Prevalence (n/N)** | **aOR# (95% CI), p-value** |
| Average | 35.2%  (438/1245) | - | 36.0%  (448/1245) | - |
| Low | 24.6%  (167/679) | **0.65 (0.52, 0.83),**  **p<0.001** | 28.0%  (190/679) | 0.82 (0.66, 1.03),  p=0.085 |
| Child high-decreasing | 40.8%  (144/353) | **1.32 (1.01, 1.73),**  **p=0.040** | 38.5%  (136/353) | 1.22 (0.93, 1.59),  p=0.146 |
| Child average-increasing | 66.7%  (86/129) | **3.86 (2.50, 5.98),**  **p<0.001** | 58.1%  (75/129) | **3.06 (2.00, 4.69),**  **p<0.001** |
| High | 52.2%  (36/69) | **2.48 (1.43, 4.28),**  **p=0.001** | 37.7%  (26/69) | 1.46 (0.85, 2.53),  p=0.172 |

# Odds Ratios and 95% Confidence Intervals from logistic regression models adjusting for adult physical activity, childhood second-hand smoking, alcohol consumption, and smoking.

Abbreviations: aOR=Odds ratio; BMI=Body mass index; CI = Confidence interval; OSA=Obstructive sleep apnoea

The estimates were in bold if p-value<0.05.

**Table S3. The association of BMI trajectories with probable OSA defined by STOP-Bang questionnaires in participants with BMI**$\leq$ **35kg/m^2^ (n=2215).**

|  | **STOP-Bang questionnaire** | |
| --- | --- | --- |
| **BMI-trajectories** | **Prevalence**  **(n/N)** | **aOR# (95% CI),**  **p-value** |
| Average | 11.2%  (130/1164) | - |
| Low | 8.9%  (59/665) | 0.76 (0.53, 1.10), p=0.142 |
| Child high-decreasing | 11.5%  (35/304) | 1.11 (0.73, 1.70), p=0.626 |
| Child average-increasing | 20.0% (10/50) | **2.58 (1.14, 5.87), p=0.023** |
| High | 12.5%  (4/32) | 1.22 (0.36, 4.16), p=0.749 |

# Odds Ratios and 95% Confidence Intervals from logistic regression models adjusting for adult physical activity, childhood second-hand smoking, alcohol consumption, and smoking.

Abbreviations: aOR=Odds ratio; BMI=Body mass index; CI = Confidence interval; OSA=Obstructive sleep apnoea

The estimates were in bold if p-value<0.05.

**Table S4. Effect modification for the association between BMI trajectories and probable OSA defined using 3 screening questionnaires.**

| ***Potential effect modifier*** | **LRT p-value** | | |
| --- | --- | --- | --- |
|  | **STOP-Bang** | **Berlin** | **OSA-50** |
| *Sex (male vs female)* | 0.1782 | 0.1092 | 0.3103 |
| *Smoking (current vs not current)* | 0.7743 | 0.9093 | 0.0493* |
| *Asthma (current vs not current)* | 0.0337* | 0.2919 | 0.7169 |
| *Diet (quartiles)* | 0.1796 | 0.3017 | 0.2869 |
| *Physical activities (low vs moderate to high)* | 0.1814 | 0.4675 | 0.2598 |

*P-value<0.1

Abbreviations: BMI=Body mass index; OSA=Obstructive sleep apnoea

**Table S5. The associations of childhood BMI z-score at 7 years with probable OSA (defined by STOP-Bang, OSA-50 and Berlin questionnaires) and clinically significant diagnosed OSA.**

|  | **STOP-Bang** | | **Berlin** | | **OSA-50** | | **Clinically significant diagnosed OSA&** |
| --- | --- | --- | --- | --- | --- | --- | --- |
|  | **Prevalence**  **(n/N)** | **aOR# (95% CI),**  **p-value** | **Prevalence**  **(n/N)** | **aOR# (95% CI),**  **p-value** | **Prevalence**  **(n/N)** | **aOR# (95% CI), p-value** | **aOR# (95% CI),**  **p-value** |
| Normal weight | 14.3%  (314/2197) | reference | 34.7%  (763/2197) | reference | 35.2%  (776/2197) | reference | reference |
| Overweight | 14.0%  (31/221) | 1.40 (0.90, 2.18)  p=0.133 | 41.6% (92/221) | **1.39 (1.04, 1.85)**  **p=0.026** | 35.8%  (79/221) | 1.13 (0.83, 1.52)  p=0.438 | 1.55 (0.93, 2.58), p=0.092 |
| Obesity | 10.0%  (2/20) | 1.46 (0.30, 7.22)  p=0.643 | 25%  (5/20) | 0.68 (0.25, 1.89)  p=0.461 | 20%  (4/20) | 0.55 (0.18, 1.68)  p=0.298 | 1.47 (0.41, 5.33), p=0.556 |

& The result for clinically significant diagnosed OSA is based on multiply imputed data.

# Odds Ratios and 95% confidence Intervals from logistic regression models adjusted for childhood second-hand smoking, gender.

Abbreviations: aOR = Adjusted odds ratio; BMI=Body mass index; BQ=Berlin questionnaires; CI = Confidence interval; OSA=Obstructive sleep apnoea

The estimates were in bold if p-value<0.05.

**Table S6. The associations of BMI z-score at 43 years with probable OSA (defined by STOP-Bang, OSA-50 and Berlin questionnaires) and clinically significant diagnosed OSA.**

|  | **STOP-Bang** | | **Berlin** | | **OSA-50** | | **Clinically significant diagnosed OSA&** |
| --- | --- | --- | --- | --- | --- | --- | --- |
|  | **Prevalence**  **(n/N)** | **aOR# (95% CI),**  **p-value** | **Prevalence**  **(n/N)** | **aOR# (95% CI),**  **p-value** | **Prevalence**  **(n/N)** | **aOR# (95% CI), p-value** | **aOR# (95% CI),**  **p-value** |
| Normal weight (18.5$\leq$BMI<25) | 4.5%  (48/1060) | reference | 18.5%  (196/1060) | reference | 20.6%  (218/1060) | reference | reference |
| Under weight  (BMI<18.5) | 2.9%  (1/34) | 0.80 (0.11, 6.12)  p=0.834 | 26.5%  (9/34) | 1.65 (0.71, 3.84)  p=0.243 | 32.4%  (11/34) | 2.17 (1.00, 4.73)  p=0.050 | 0.57 (0.14, 2.30), p=0.432 |
| Overweight  (25$\leq$BMI<30) | **17.4%**  **(165/947)** | **5.14 (3.54, 7.48)**  **p<0.001** | **40.3%**  **(382/947)** | **3.05 (2.44, 3.82)**  **p<0.001** | **41.8%**  **(396/947)** | **2.75 (2.22, 3.42)**  **p<0.001** | **2.47 (1.70, 3.59), p<0.001** |
| Obesity  (BMI$\geq$30) | **31.6%**  **(137/434)** | **11.70 (7.77, 17.61)**  **p<0.001** | **65.4%**  **(284/434)** | **9.03 (6.78, 12.02)**  **p<0.001** | **57.6%**  **(250/434)** | **5.63 (4.27, 7.40)**  **p<0.001** | **4.86 (2.94, 8.05), p<0.001** |

& The result for clinically significant diagnosed OSA is based on multiply imputed data.

# Odds Ratios and 95% confidence Intervals from logistic regression models adjusted for adulthood physical activities, childhood BMI, alcohol consumption, smoking.

Abbreviations: aOR = Adjusted odds ratio; BMI=Body mass index; BQ=Berlin questionnaires; CI = Confidence interval; OSA=Obstructive sleep apnoea

The estimates were in bold if p-value<0.05.


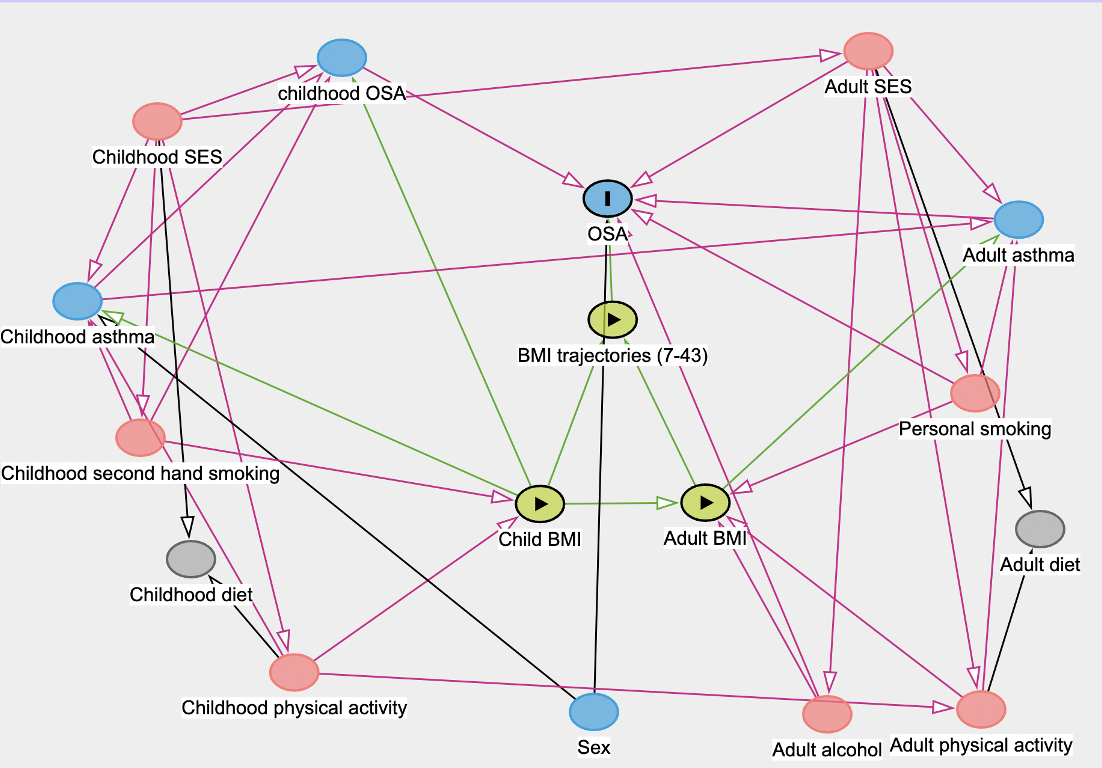


**Figure S1. The directed acyclic graph (DAG) of possible causal associations.** The minimal set of confounders included childhood physical activity (no data available), adult physical activity, adult smoking, childhood second-hand smoking status, and adult alcohol consumption


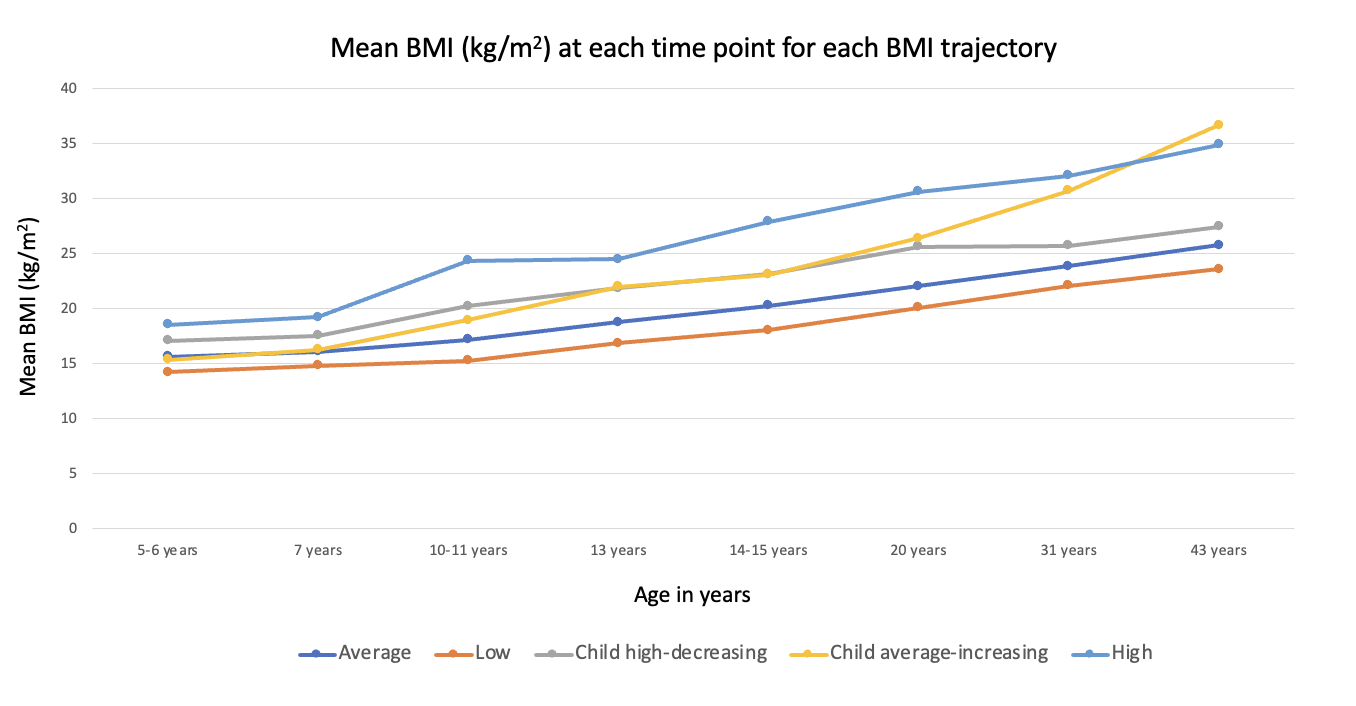


**Figure S2. Line plot of mean body mass index at each time point for each trajectory.**

Abbreviations: BMI=body mass index.

**References**

1. Van Buuren S. Flexible imputation of missing data: CRC press; 2018.

2. Lee KJ, Simpson JA. Introduction to multiple imputation for dealing with missing data. Respirology. 2014;19(2):162-7.

3. White IR, Royston P, Wood AM. Multiple imputation using chained equations: issues and guidance for practice. Statistics in medicine. 2011;30(4):377-99.

4. Li P, Stuart EA, Allison DB. Multiple imputation: a flexible tool for handling missing data. Jama. 2015;314(18):1966-7.

5. Vidmar SI, Cole TJ, Pan H. Standardizing anthropometric measures in children and adolescents with functions for egen: update. The Stata Journal. 2013;13(2):366-78.
