# Supplementary material for: Early‐to‐Midlife Body Mass Index Trajectories and Obstructive Sleep Apnoea Risk 10 Years Later
Source: Respirology. 2025 Feb 11;30(5):435–45. doi: 10.1111/resp.70002 (PMC12060751; doi:10.1111/resp.70002)
Supplement: Supplementary file 2 — Visual Abstract Early‐to‐midlife body mass index (BMI) trajectories and obstructive sleep apnoea (OSA) risk 10 years later [file RESP-30-435-s002.pdf]

Early-to-midlife body mass index (BMI) trajectories and obstructive sleep apnoea (OSA) risk 10 years later

Exposure (BMI trajectories)

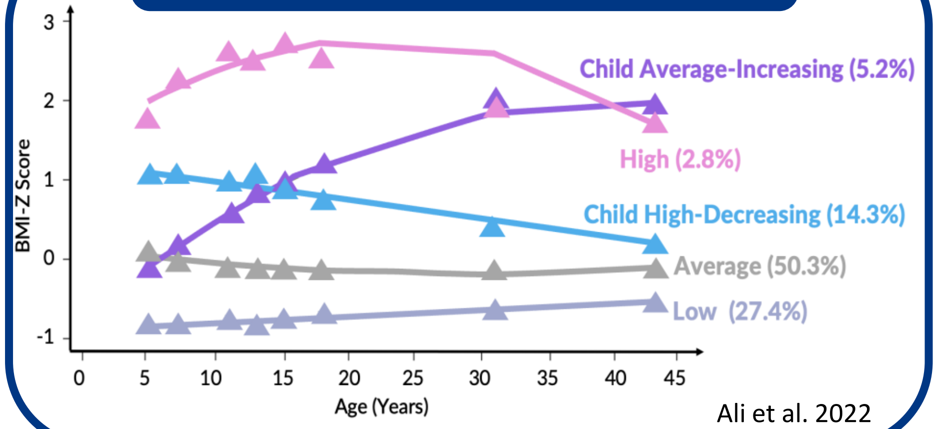

Ali et al. 2022

Outcome (OSA)

- ❖ **Probable OSA defined using questionnaires**
  - **STOP-Bang**
  - **Berlin**
  - **OSA-50**

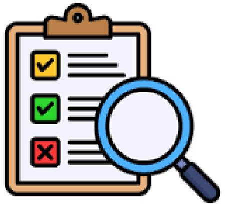

- ❖ **Clinically significant diagnosed OSA**

Key Findings & Implications

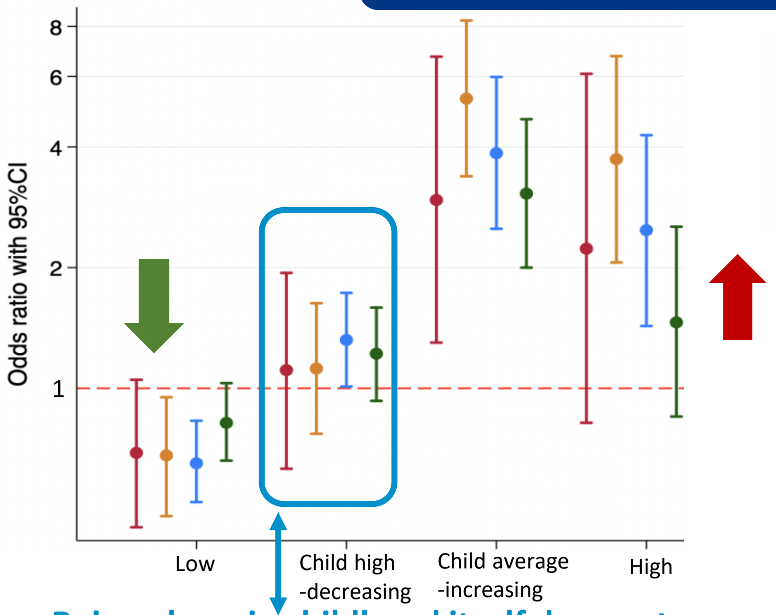

- **Clinically significant diagnosed OSA**
- **STOP-Bang defined probable OSA**
- **Berlin defined probable OSA**
- **OSA-50 defined probable OSA**

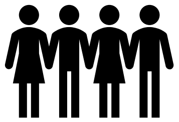

Sample

N=2475 in TAHS

- **Being obese in childhood itself does not necessarily increase the risk of adulthood OSA, if weight is subsequently reduced !**
- **Physicians and the public should be aware of the potential risk of OSA in middle-aged adults when BMI is persistently high or continuously increasing from childhood to mid-40s.**
- **Maintaining a normal weight throughout life would be beneficial in reducing the risk of OSA.**

Respirology

Early-to-midlife body mass index trajectories and obstructive sleep apnoea risk 10 years later.

QIAN et al. 2025; DOI: 10.1111/resp.70002
